# Gastroduodenal Tuberculosis Presenting as a Gastric Outlet Obstruction: A Case Report

**DOI:** 10.7759/cureus.49436

**Published:** 2023-11-26

**Authors:** Abdul Majeed Maliyakkal, Vamanjore A Naushad, Nabeel M Shaath, Hanee S Valiyakath, Khalifa L Farfar, Asad M Mohammed, Mustafa Ahmed

**Affiliations:** 1 Department of Medicine, Hamad Medical Corporation, Doha, QAT; 2 Clinical Department, College of Medicine, QU Health, Qatar University, Doha, QAT; 3 Clinical Medicine, Weill Cornell Medicine, Doha, QAT; 4 Department of Gastroenterology, Hamad Medical Corporation, Doha, QAT; 5 Department of Radiology, Hamad Medical Corporation, Doha, QAT

**Keywords:** peripancreatic lymph node, endoscopic ultrasound, gastric outlet obstruction, gastroduodenal tuberculosis, abdominal tuberculosis

## Abstract

Abdominal tuberculosis (TB) can affect any part of the gastrointestinal tract, solid organs, peritoneum, or lymph nodes. The diagnosis of abdominal TB is usually delayed due to a lack of specific clinical signs and symptoms and the mimicking of other intra-abdominal diseases. We present a case of gastroduodenal tuberculosis with peripancreatic lymph node involvement presented as a gastric outlet obstruction that was treated conservatively with anti-tuberculosis medications.

## Introduction

Abdominal tuberculosis accounts for about 11-16% of all tuberculosis (TB) cases [1,2]. It can affect any part of the gastrointestinal (GI) tract, solid organs, peritoneum, or lymph nodes. Due to the lack of specific clinical signs and symptoms and the mimicking of other intra-abdominal diseases, the diagnosis of abdominal TB is usually delayed [3]. Fifteen percent to 25% of patients with abdominal TB will have a concomitant involvement of the lungs [4,5]. In many countries where TB is not endemic, due to a surge in the migrant population or a rise in the incidence of HIV infections, there is an increase in the incidence of TB in these nations [6-8]. Gastrointestinal tuberculosis (GI-TB) is one of the forms of abdominal TB, the others being visceral TB, peritoneal TB, and tuberculous lymphadenopathy. Gastric outlet obstruction (GOO) is commonly caused by peptic ulcer disease (PUD) and gastric malignancy; however, with the introduction of proton pump inhibitors and Helicobacter eradication therapy, the incidence of PUD-related GOO has been on the decline. Other causes of GOO include large gastric polyps, Crohn's disease, post-gastric surgery complications, caustic ingestion, pancreatitis-related fluid collection, and gastric bezoars. GOO is one of the rare presentations of gastroduodenal tuberculosis. Here, we present a case of gastroduodenal tuberculosis presenting as a gastric outlet obstruction, which was treated conservatively.

## Case presentation

A 49-year-old Nepalese gentleman with no prior significant medical history was admitted to the hospital with complaints of recurring bouts of upper abdominal pain and vomiting of 10 months’ duration that had worsened three days prior to the admission. He also reported to have decreased appetite and a loss of weight of 10 kilograms. History of fever, cough, alteration in bowel habits, hematemesis, or melena was negative. There was no family history of tuberculosis or cancer. His general and systemic examination was unremarkable except for dehydration and underweight (BMI 17.9 kg/m^2^). The basic hematological and metabolic profile on admission was unremarkable. HIV serology was negative and interferon-γ release assay (QuantiFERON-TB Gold™; Qiagen® Inc., Hilden, Germany) was positive (Table 1).

**Table 1:** Results of laboratory investigations

Tests	Results	Normal reference range
WBC	8.2	(4 – 10 × 10^9^/L)
Neutrophils	4.0	(2-7 × 10^9^/L)
Hemoglobin	14.4	(12–15 gm/dL)
Platelet	293	(150-400 × 10^3 ^/ µL)
CRP	1	(0.0-5.0 mg/L)
Serum sodium	136	(133-146 mmol/L)
Potassium	4.8	(3.5-5.3 mmol/L)
Chloride	103.9	(95-108 mmol/L)
Bicarbonate	27.8	(22-29 mmol/L)
Blood urea	4.9	(2.5-7.8 mmol/L)
Creatinine	97	(62-106umol/L)
Random Glucose	4.5	(3.3-5.5 mmol/L)
Serum lipase	23	(8-78 U/L)
Lactate	0.6	(0.5-2.2 mmol/L)
Total bilirubin	8.5	(3.5-24 µmol/L)
AST	18	(0 - 30 U/L)
ALT	14	(0 - 31 U/L)
ALP	73	(45-129 U/L)
HbA1c	5.4	(4.8 – 6.0 %)
HIV serology	Negative	
Quantiferon TB test	Positive	

**Figure 1:**
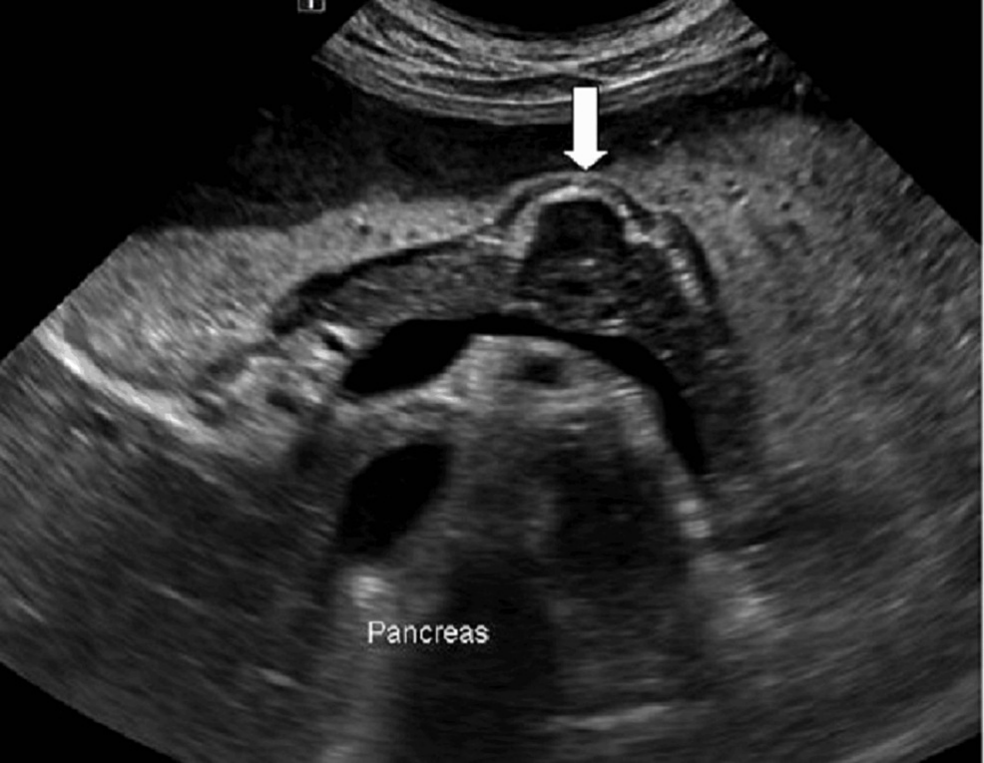
Ultrasound of the abdomen showing a distended stomach filled with fluid/food matter and hypoechoic lesion (white arrow) in the epigastric region related to the body of the pancreas

The chest radiograph was normal. Ultrasound examination of the abdomen revealed a distended stomach filled with fluid/food matter and a hypoechoic lesion in the epigastric region related to the body of the pancreas (Figure 1).

**Figure 2:**
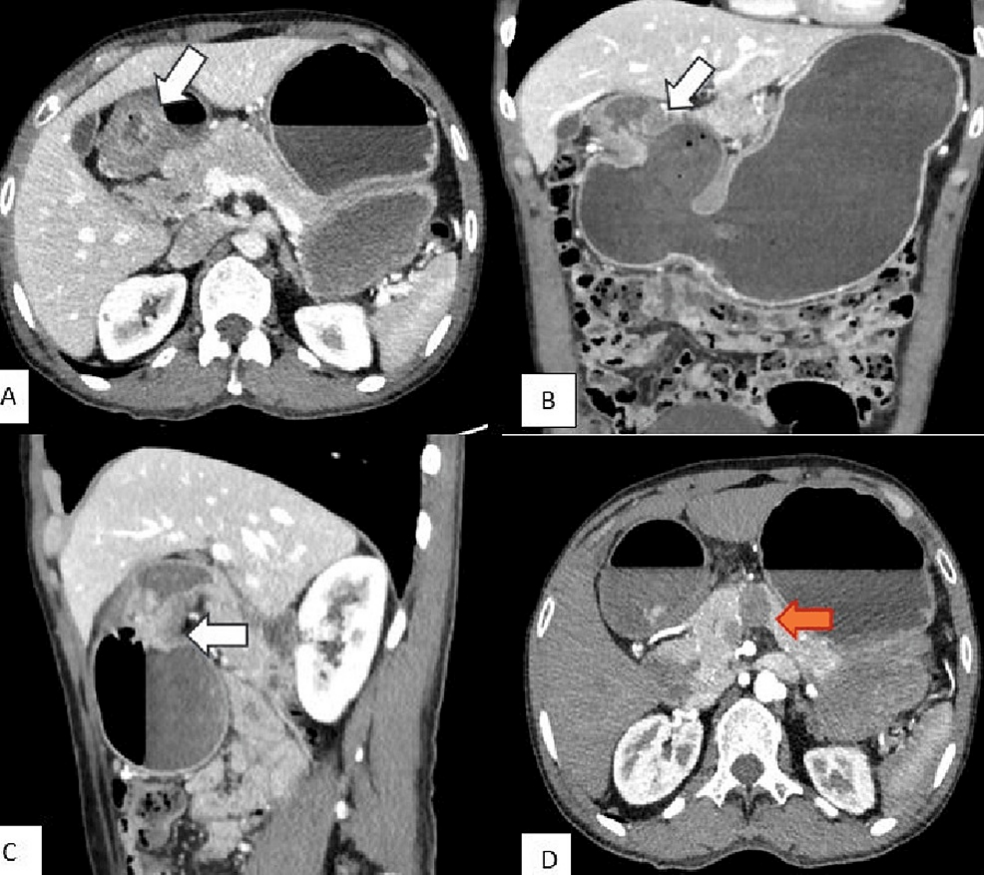
Computed tomography (CT) scan of the abdomen (A-axial, B-coronal, C-sagittal views): showing diffuse dilatation of the stomach up to the antropyloric level with abrupt narrowing in the pyloric region (white arrow) over a length of approximately 2.4 cm caused by circumferential and asymmetric wall thickening. D – Axial view: showing a large hypodense peri-pancreatic lymph node measuring 22 mm x 17 mm (red arrow).

Further evaluation by computed tomography (CT) scan of the abdomen showed diffuse dilatation of the stomach up to the antropyloric level. Abrupt narrowing was seen in the pyloric region over a length of approximately 2.4 cm caused by circumferential and asymmetric wall thickening. A large hypodense peripancreatic lymph node was also noted, measuring 22 mm x 17 mm (Figure 2).

**Figure 3:**
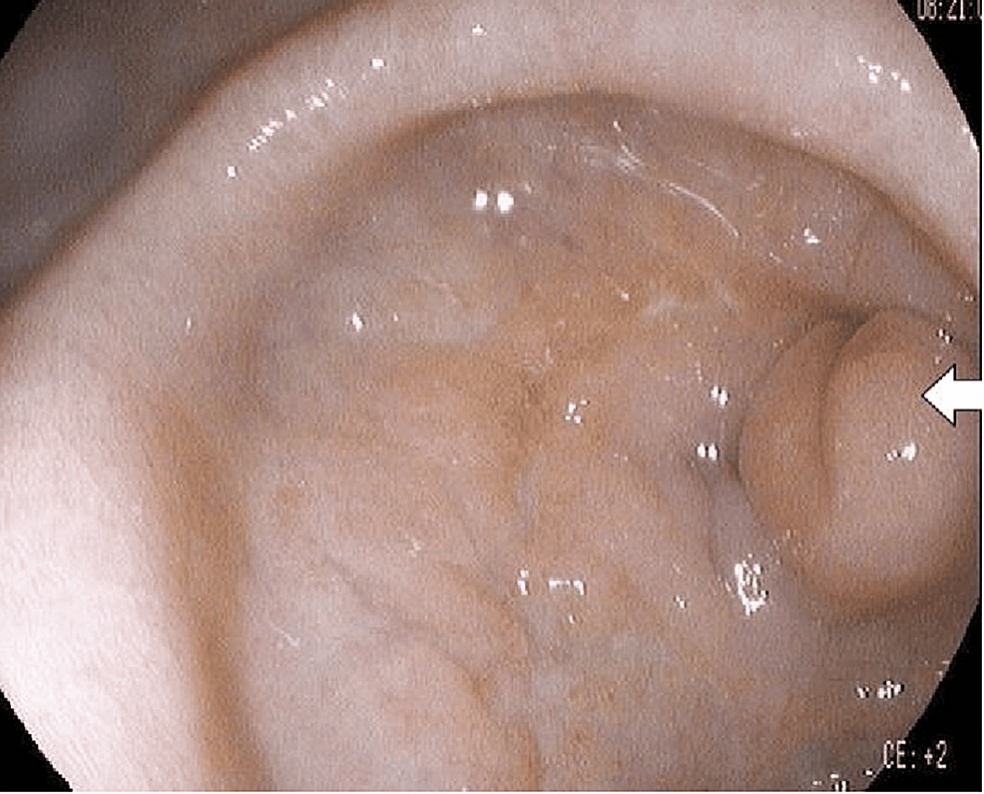
Endoscopic picture showing the pre-pyloric scar and deformed pylorus

The CT scan features were suggestive of gastric outlet obstruction, and given the circumferential wall thickening in the antropyloric region and a large peripancreatic lymph node, raised the suspicion of upper GI malignancy. He was treated conservatively with intravenous fluids, proton pump inhibitors, and antiemetics pending further evaluation. An upper GI endoscopy was performed on the following day, which showed a healing pre-pyloric ulcer with a scar (Figure 3).

**Figure 4:**
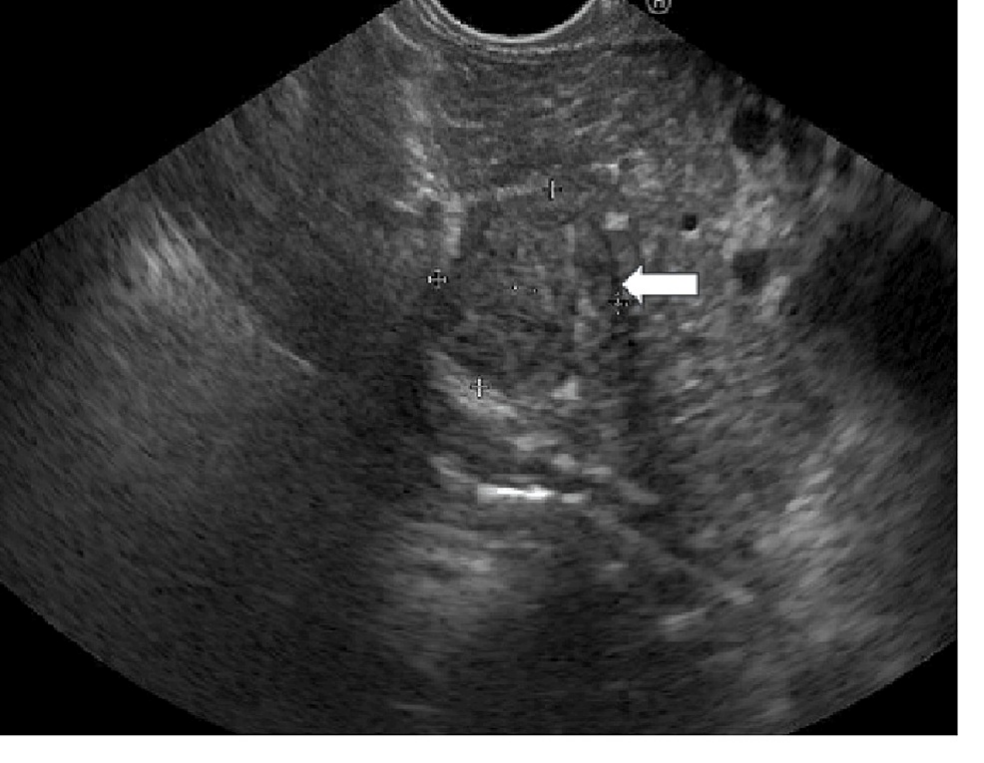
Endoscopic ultrasound showing a hypoechoic mass lesion of 19x18 mm (arrow) between the pancreatic neck and gastric wall

The campylobacter-like organism test (CLO test) for Helicobacter pylori (H. pylori) was positive. Endoscopic ultrasound (EUS) revealed a hypoechoic mass lesion with cavitation (15x18 mm in diameter) between the pancreatic neck and gastric wall (Figure 4).

No mass lesion was noted in the pancreas. The pancreatic duct was normal in size. Under EUS guidance, fine-needle aspiration of the lesion was performed. The polymerase chain reaction test (PCR) of the aspirate for Mycobacterium tuberculosis (MTB) was positive; however, the culture was negative for MTB. Cytopathology revealed abundant necrotizing debris and the stain for acid-fast bacilli (AFB) was negative. Evaluation for pulmonary tuberculosis was negative.

The case was treated as gastroduodenal and peripancreatic lymph node tuberculosis with features of gastric outlet obstruction. The ulcer in the antropyloric region was attributed to being of tuberculosis.

First-line antituberculosis therapy containing rifampicin (600 mg/day) and isoniazid (300 mg/day) for six months, ethambutol (1100 mg/day), and pyrazinamide (1600 mg/day) for two months was started along with pyridoxine (40 mg/day). In view of the positive CLO test, quadruple therapy (tripotassium dicitrato bismuthate 120 mg q6hr, metronidazole 250 mg q6hr, tetracycline 250 mg, and pantoprazole 40 mg twice daily) for two weeks was also prescribed.

**Figure 5:**
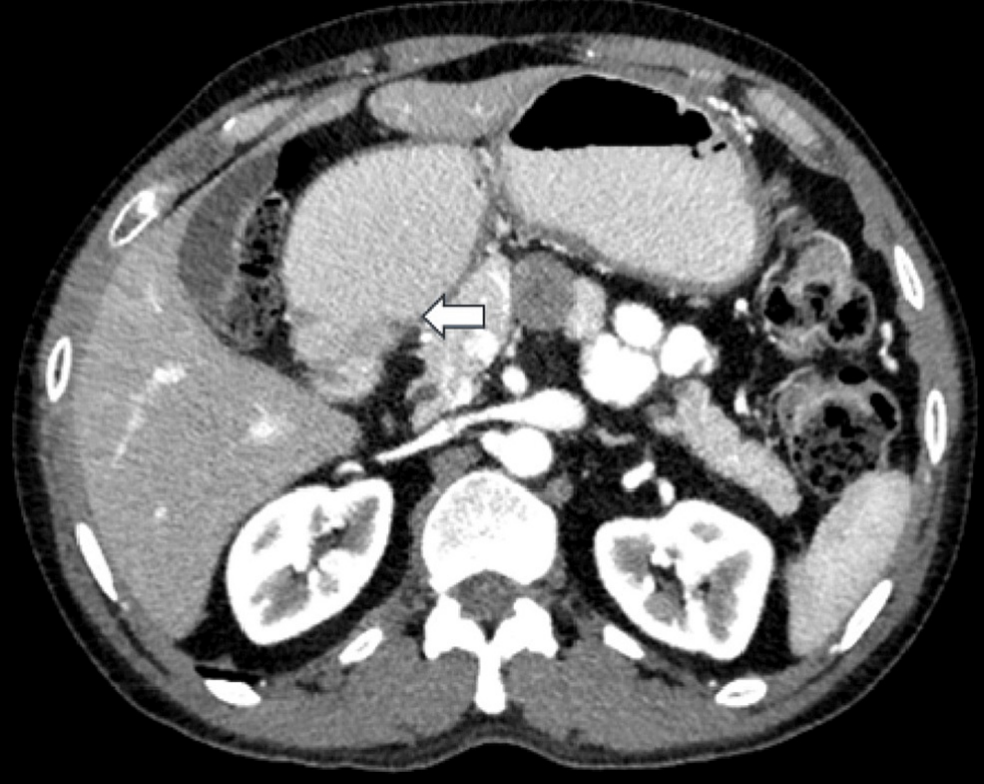
Repeat CT scan of the abdomen with contrast (axial view) showing regression of circumferential and asymmetric wall thickening at the pyloric region (arrow) and regression of dilatation of the stomach

The patient was discharged home after eight days of hospitalization. On follow-up after two months, his symptoms showed remarkable improvement with subsiding abdominal pain, improved appetite, and weight gain. Upon completion of anti-TB treatment after six months, his body weight increased by nine kilograms, and he was completely asymptomatic. A repeat CT scan of the abdomen with contrast revealed regression of circumferential and asymmetric wall thickening at the pyloric region and regression of dilatation of the stomach (Figure 5). Redemonstration peripancreatic hypodense lymph node shows no appreciable interval change.

## Discussion

In gastrointestinal tuberculosis, the ileocecal region is the most common site involved, followed by the jejunum and colon. Jejunal and ileocecal TB account for 64% of cases of GI-TB [9]. The esophagus, stomach, and duodenum are rare sites of GI-TB. Primary involvement of the stomach (gastric TB) and duodenum is very rare. Primary gastric TB is seen in 0.4-2%, whereas primary duodenal TB is seen in 2-2.5% of all GI-TB cases [10,11].

The reason for the low incidence of involvement of the stomach in TB is multifactorial. Bactericidal properties of gastric acid, thick and intact gastric mucosa, and scarcity of lymphoid tissues in gastric mucosa all might be the cause for the low rate of involvement [12]. Treatment with H2 blockers increases the risk of gastroduodenal TB (GD-TB) [13,14].

Gastric TB more commonly involves the lesser curvature and pylorus as ulcerating lesions [15]. It can also present with hypertrophic variety, miliary tubercle, and pyloric stenosis [12]. Gastric TB is usually associated with TB lymphadenitis. In the present case, an ulcerative lesion was seen in the pre-pyloric area, along with the involvement of the peripancreatic lymph node. 

In the case of primary duodenal TB, the third part of the duodenum is the most common site involved. Duodenal involvement can be intrinsic/extrinsic or both [16]. The extrinsic form is the most common variety and is secondary to lymphadenopathy in the C-loop of the duodenum. The intrinsic variety can be ulcerative, hypertrophic, or ulcero-hypertrophic. Duodenal TB can lead to stricture or fistula formation.

The modes of involvement of GI-TB occur by ingestion of infected sputum (secondary TB), ingestion of infected milk or food (primary TB), hematogenous spread from distant TB focus, or contiguous spread from infected adjacent foci through lymphatic channel [17,18]. In our case the evaluation for extra-abdominal TB was negative, and we believe it was primary gastric TB with involvement of the peripancreatic lymph node.

The clinical features of gastro-duodenal TB are nonspecific. Most present with epigastric pain, weight loss, and fever. In some cases, a gastric outlet obstruction can be the presenting feature. The differential diagnosis of gastric TB includes carcinoma and lymphoma. A retrospective review of 23 histologically proven gastro-duodenal TB in a single center in India by Yannam et al. reported that vomiting (60.8%) and epigastric pain (56.5%) were the most common presenting symptoms in their patients. Furthermore, 14 (61%) patients had features of gastric outlet obstruction. The duodenal stricture was the cause of obstruction in 12 patients; whereas, only two had pyloric stenosis. Interestingly, none of their patients were HIV positive, whereas four patients had diabetes mellitus [13]. Our patient had primarily epigastric pain and vomiting with features of gastric outlet obstruction. Furthermore, the serological test for HIV was negative. A review of 49 patients with duodenal TB by Gleason et al. also reported pain (73%) and vomiting (55%) as the most common presenting symptoms [19].

The diagnosis of gastro-duodenal TB is often missed due to a paucity of disease-specific clinical symptoms and signs. Even though the histopathological diagnosis of the gastro-duodenal biopsy specimen by endoscopy is the gold standard for the diagnosis of gastro-duodenal TB, this may not be feasible always. Furthermore, even in biopsy specimens, granulomatous lesions are rarely found because of the submucosal location of these lesions. In a review of 27 patients with duodenal TB who underwent endoscopic biopsy, granulomas were found only in seven of them [20]. Similarly, Yannam et al. in their review had positive biopsies in 2 out of 20 patients [13]. Even though the detection or culture of AFB from the biopsy specimen may improve the rate of diagnosis, the yield is usually low. CT scan of the abdomen might show thickening of the gastric or duodenal wall with or without local lymphadenopathy [13]. In the present case, the CT scan abdomen showed features of gastric outlet obstruction with circumferential wall thickening in the antropyloric region and a large peripancreatic lymph node. PCR from the FNA specimen of the peripancreatic lymph node was positive for mycobacterium tuberculosis.

The complications of GD-TB include gastric outlet obstruction, hemorrhage, and perforation. GD-TB with gastric outlet obstruction can lead to increased morbidity. Even though in the present case patient’s symptoms improved with antituberculosis treatment without any surgical intervention this need not always be the case. A review by Yannam et al. reported that 12 out of 14 patients with gastric outlet obstruction due to tuberculosis underwent truncal vagotomy and gastro jejunostomy, with or without feeding jejunostomy. All their patients received a standard four-drug regimen of anti-tuberculosis treatment (ATT) [13].

In our patient, after six months upon completion of the ATT, a repeat CT scan of the abdomen with contrast revealed regression of circumferential and asymmetric wall thickening at the pyloric region and regression of dilatation of the stomach. Redemonstration peripancreatic hypodense lymph node shows no appreciable interval change. Previous studies have reported similar findings, where CT scan images failed to show mass regression even after receiving a full course of ATT despite complete symptom resolution. A retrospective study of 35 patients of peripancreatic TB evaluating the treatment response based on computed tomography criteria showed complete response in 10 patients (28.6%), partial response in 23 patients (65.7%), and stable disease in 1 patient (2.9%) [21]. Garcia et al., in their case report, described a similar case of primary peripancreatic lymph node TB, which had complete symptom resolution although lesion size remained unchanged even after receiving a full course of ATT [22].

## Conclusions

Abdominal tuberculosis presenting as a gastric outlet obstruction is extremely rare. This case describes a rare presentation of gastroduodenal and peripancreatic lymph node tuberculosis presenting as a gastric outlet obstruction that was managed conservatively with anti-TB medications, without requiring surgery. Though rare, gastroduodenal tuberculosis should be considered as one of the possible differential diagnoses in patients presenting with features of gastric outlet obstruction. EUS-guided FNA is an important tool in sampling retroperitoneal/peripancreatic lymph nodes and could help avoid a surgical biopsy.
